# A Case for Risk Stratification in Survivors of Firearm and Interpersonal Violence in the Urban Environment

**DOI:** 10.5811/westjem.2020.8.45041

**Published:** 2020-10-16

**Authors:** Garth N. Walker, Annette M. Dekker, David A. Hampton, Adesuwa Akhetuamhen, P. Quincy Moore

**Affiliations:** * Northwestern Buehler Center Health Economics and Policy and Northwestern Department of Emergency Medicine, Chicago, Illinois; † University of California, Los Angeles, Department of Emergency Medicine, Los Angeles, California; ‡ University of Chicago, Department of Surgery, Section of Trauma and Acute Care Surgery, Chicago, Illinois; § University of Chicago, Department of Medicine, Section of Emergency Medicine, Chicago, Illinois

## Abstract

The emergency department (ED) serves as the main source of care for patients who are victims of interpersonal violence. As a result, emergency physicians across the nation are at the forefront of delivering care and determining dispositions for many at-risk patients in a dynamic healthcare environment. In the majority of cases, survivors of interpersonal violence are treated and discharged based on the physical implications of the injury without consideration for risk of reinjury and the structural drivers that may be at play. Some exceptions may exist at institutions with hospital-based violence intervention programs (HVIPs). At these institutions, disposition decisions often include consideration of a patient’s risk for repeat exposure to violence. Ideally, HVIP services would be available to all survivors of interpersonal violence, but a variety of current constraints limit availability. Here we offer a scoping review of HVIPs and our perspective on how risk-stratification could help emergency physicians determine which patients will benefit most from HVIP services and potentially reduce re-injury secondary to interpersonal violence.

## INTRODUCTION

Firearm and interpersonal violence have costly downstream effects that continue to burden the health of many communities across the nation. In the United States from 2006 through 2014, over 700,000 emergency department (ED) visits were related to firearm violence.^1^ In 2016 alone, approximately 37,900 deaths in the U.S. were due to firearm violence, 82% of which occurred in urban settings.^2^ Those who survive interpersonal violence are at a one in four risk of being repeat victims of interpersonal violence, also known as injury recidivism.^3^,^4^ Injury recidivism is associated with a five percent mortality rate over five years.^5^ Studies have shown that hospital-based violence intervention programs (HVIPs) are a promising step toward helping these high-risk patients.^6^–^8^ Ideally, all survivors of firearm and interpersonal violence would receive aid from a hospital-based violence intervention program. However, given resource limitations, we believe that risk stratification of interpersonal violence survivors in the ED offers the opportunity to target valuable resources to those most in need, and potentially decrease costs directly and indirectly related to interpersonal violence.

In this article, we discuss injury recidivism and HVIP in survivors of interpersonal violence, current management strategies for disposition of victims of interpersonal violence including a scoping review of hospital-based violence intervention programs, and considerations of how to improve outcomes. Ultimately, we advocate for research to develop a clinical decision tool that can be used in the emergency department to identify those at highest risk for reinjury and those that would benefit most from focused intensive intervention. In this paper, we will refer to “interpersonal violence” as a term that includes penetrating injuries and assault, but excludes intimate partner violence and self-harm. We will also use the terms “injury recidivism” and “reinjury” interchangeably to refer to repeat injuries suffered by those who were previously survivors of interpersonal violence.

## INJURY RECIDIVISM

High rates of injury recidivism have been well documented in urban settings for decades. As early as the 1980s, Henry Ford Hospital in Detroit, Michigan identified that survivors of violent trauma had a 44% rate of recurrent traumatic injury with a 5-year mortality rate of 20%.^9^ More recent studies in Baltimore, Oakland, and New York City are similarly disheartening.^3^–^5^ In Baltimore, survivors of interpersonal violence experience a 15.7% rate of injury recidivism, with the rate of subsequent mortality for survivors of penetrating trauma increasing by more than twofold for each additional instance of penetrating trauma.^4^ In New York City, patients presenting with penetrating trauma had a 27% chance of fatal injury if they had a previous encounter for penetrating trauma, compared to 3% in those who did not.^3^ In Oakland, homicide was the cause of death in 80% of gunshot victims who survived the index injury.^5^ It is clear that the circumstances that contribute to interpersonal violence put survivors at high risk of reinjury. Each presentation to the ED offers an opportunity to intervene in hopes of reducing future morbidity, mortality, and healthcare expenditures.

## CURRENT PRACTICE FOR VICTIMS OF INTERPERSONAL VIOLENCE

Despite the high rate of injury recidivism, the disposition of survivors of interpersonal violence is driven primarily by medical history, physical exam, labs, and imaging used to assess the extent of physical injury. At most institutions, the potential for repeat traumatic injury does not factor into the decision of whether or not a patient is dispositioned home or whether additional resources are indicated. Exceptions to this include an increasing number of hospitals located in cities with high rates of interpersonal violence that are pioneering HVIPs to reduce the risk of reinjury. At the majority of these institutions, HVIPs offer services to all individuals and do not tailor care based on risk of reinjury.

## METHODS

We chose a scoping review for this project to provide a preliminary overview of the existing gaps in the literature. We utilized the PRISMA-ScR checklist to adhere to methodically build and summarize our findings.

Our research question aimed to review studies that measure the impact that HVIPs have on injury recidivism. We organized our results by study design and summarize significant results and concordant discussion sections.

Our search was designed to capture primary research that explored the impact of HVIPs on injury recidivism. We explored two comprehensive libraries (Pubmed and SCOPUS) with relevant MeSH terms and keywords, i.e. “injury recidivism”, “hospital-based violence intervention programs”. One reviewer (GNW) performed a search and screening of all abstracts identified in PubMed. A second reviewer (AMD) performed a search and screening of all abstracts identified in SCOPUS. We restricted search to English language, United States, and time period of January 2000 to December 2018. We then built an Endnote library that included all of the selected research articles. To ensure we extracted the appropriate research for our paper, we examined the bibliography of all selected papers accordingly and added any additional findings.

**Population Health Research Capsule**

What do we already know about this issue?

*Survivors of interpersonal violence are more likely to be repeat victims of violence with high rates of associated mortality. Risk-stratification tools have helped determine who receives limited resources in other disease states*.

What was the research question?

*We examined the current literature on hospital-based violence intervention programs (HVIP) to understand their role in reducing injury recidivism and explore the role of risk-stratification tools to predict reinjury*.

What was the major finding of the study?

The effect of HVIPs is promising but inconclusive. Longitudinal research, risk

*tools, and trainee education may improve their effectiveness*.

How does this improve population health?

*A risk-stratification tool that identifies the patients who would most benefit from HVIP services would mitigate the downstream implications – physical, mental, and financial – for patients as well as their communities*.

We included primary research papers that reported implementation of hospital-based violence intervention programs through the ED or hospital with a defined patient population, intervention, and follow-up period. Our outcome measures included either injury recidivism or other potential markers of experience with violence including attitudes toward violence, criminal offenses, and additional parameters focused on future injury reduction.

The primary author (GNW) reviewed the title and abstract extracted from PUBMED of each article to assess relevance to our research question. AMD reviewed title and abstracts extracted from SCOPUS. Both AMD and GNW each reviewed the full text to assess the methodology and strength of each study. Studies were extracted from SCOPUS by AMD then abstracts reviewed separately by GNW.

For each study, we tabulated the year of publication, authors, sample size, location, intervention, design, follow-up, and primary and secondary outcomes. (See Table 1) We summarized the data using common themes related to the research question.

## RESULTS

**Figure 1:**
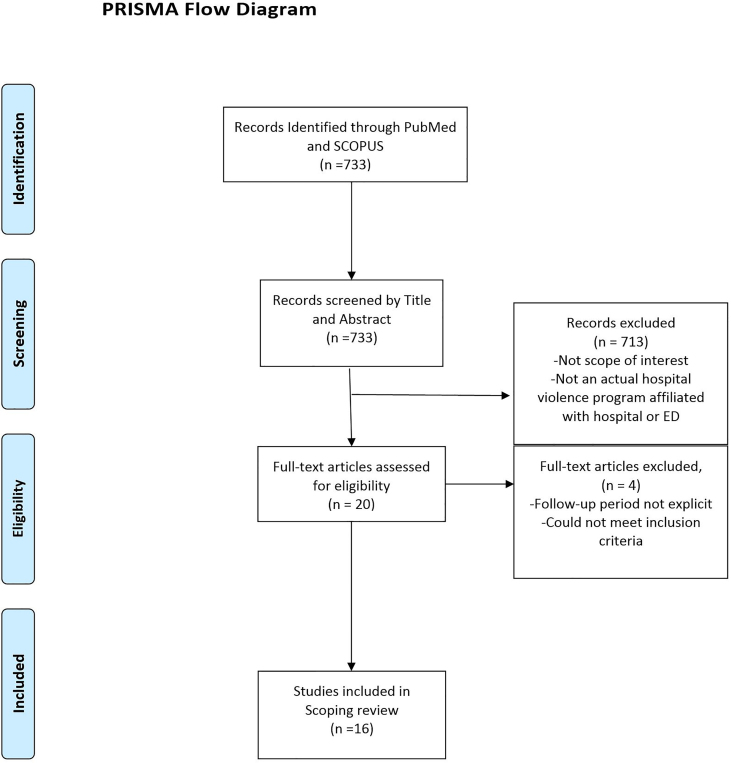
The PRIMSA diagram details our search and selection process applied during the overview. *ED*, emergency department.

We reviewed 727 publications, of which 16 articles met our inclusion criteria. (see PRISMA flowchart Figure 1) The age range of patient participants differed among programs from pediatric patients only^10^,^11^ to those eighteen years and older.^12^–^15^ All studies except one had a minimum inclusion criterion that participants had suffered intentional violent injuries. The exception was Operation Peace Works in California, which was based on referrals from the criminal justice system.^16^ A few studies focused more specifically on those who suffered violent injuries and had an additional risk factor, such as involvement with the criminal justice system^15^,^16^ or admission to the hospital.^14^ Only one program, the Wraparound Project (WAP) at San Francisco General Hospital, focused interventions on individuals determined to be at high risk for reinjury.^17^,^18^ This determination of high risk for reinjury was based on structured case-manager screening assessments including, but not limited to, physical signs, social cues, emotional volatility, prior exposure to violence, and unstable family situations.^17^,^18^

Specific violence intervention strategies also differed. A few sites utlized brief interventions that were delivered via electronic-mail or performed by an in-person therapist such as SaferFlint Teens in Flint, Michigan,^19^–^21^ or through telephone-based parent education in the Minneapolis metropolitan area.^10^ Other interventions used focused case management for several months.^22^–^25^ Finally, several programs, including Youth ALIVE! in Oakland, California and Project Prescription Hope in Indianapolis, Indiana, provided intensive interventions that included personnel with specialized training, peer support, education opportunities, employment options, and legal services.^12^,^13^,^26^–^28^

Nine HVIPs included injury recidivism as an outcome measure. Four programs found no statistically significant change in injury recidivism after their intervention.^11^,^22^,^26^,^27^,^29^ A prospective cohort study of the Project Prescription Hope intervention in Indianapolis found a reduction in injury recidivism from 8.7% to 2.9%.^12^ Analogously, a retrospective cohort study of WAP data demonstrated a reduction in injury recidivism from 16% to 4% in the intervention group.^17^ Lastly, three randomized control trials found reductions in injury recidivism (control group vs intervention), including: 1) the Violence Intervention Project (VIP) in Baltimore, Maryland (35% vs. 5%^15^); 2) Within Our Reach in Chicago, Illinois (20.3% vs 8.1%^24^,^25^); and 3) telephone-based parenting education in the Minneapolis metropolitan area (OR: 0.2 95% CI: 0.06–0.75^8^–^10^).

Secondary HVIP outcomes were also assessed. HVIP participants were found to have lower aggression scores,^10^,^11^,^19^,^20^ crime rates,^26^,^27^ and associated financial burden^15^,^28^ compared to control groups. Two studies that assessed the cost effectiveness of Youth ALIVE! found the program directly contributed to a significant municipal budget savings. The first estimated a $750,000 to $1.5 million annual savings based on juvenile detention centers cost reduction.^27^ The second found an incremental cost effectiveness of $2,491 per person due to injury recidivism reduction.^28^ The Baltimore program found similar cost savings, including a reduction in costs associated with incarceration ($2 million control group vs $500,000 intervention group), hospitalization ($736,000 control group vs $1380,000 intervention group) and unemployment (80% control group vs 18% intervention group).^15^ Finally, a cost-effectiveness analysis of WAP suggested health benefits of 24 quality-adjusted life years (QALYs) and a $4,100 savings when implemented for 100 individuals.^30^

## DISCUSSION

### What is Missing? The Case for Stratification

Hospital-based violence intervention programs have a significant impact on injury recidivism and other outcomes in a number of cities across the United States. It is possible that all survivors of interpersonal violence would benefit from participation in a violence intervention program. While studies suggest a reduction in both mortality from recurrent trauma as well as associated costs, the logistical and financial barriers to implementing HVIPs are high. First, the interventions are intensive and long lasting, following patients for months to years after their initial injury. Second, the majority of traumas occur during weekends and nights, making it challenging to provide appropriate counseling in the ED.^31^,^32^ Third, with frequent ED and hospital overcrowding, boarding or admitting all patients to facilitate further intervention creates barriers that may preclude inclusion of all patients.^33^,^34^ Finally, the rate of follow-up in this patient population is notoriously low, making delayed intervention during follow-up appointments unlikely to succeed.^35^

Wide implementation of a broadly inclusive violence intervention program should remain the goal. Well-resourced programs based in the ED can be helpful in aiding successful case management or social tools for patients at risk for injury recidivism. In the absence of such a program, however, we recognize the need for targeted use of resources. In order to make the most impactful use of available resources, EPs need to be able to identify those who are at the highest risk of repeat injury in real time that evaluate patients risk holistically in the context of social and structural factors related to race, gender, and socioeconomic variables.

### Development of a Risk Stratification Tool

The development of a risk stratification tool requires: 1) identification of risk factors for reinjury; 2) internal validation; 3) external validation; and 4) feasibility and implementation studies. Based on clinical experience and existing medical literature, criteria would need to be identified that are both predictive of injury recidivism and practically implementable in the ED by physicians or other staff members that are found in an average ED. Approaches that require intensive inpatient or specialized case management interventions will be severely limited in their generalizability.

Literature suggest that certain social determinants of health and structural drivers such as: 1) male gender; 2) black race; 3) low socioeconomic status; 4) zip code; and 5) uninsurance/Medicaid, are risk factors for injury recidivism.^36^–^39^ A study based in Oakland, California that followed survivors of interpersonal violence ages 12–24 found that independent predictors of violent injury recidivism included male gender (OR: 2; 95% CI: 1.06–3.80; p = 0.03), black race (OR: 2.1; 95% CI: 1.44–3.06; p < 0.001), and living in the lowest zip code socioeconomic quartile (OR: 1.59; 95% CI 1.12–2.25; p = 0.01).^37^ This was also demonstrated for individual survivors of firearm injury (OR:1.67; 95% CI: 1.12–2.50; p = 0.01).^37^ Similarly, a Florida study investigating injury recidivism found independent predictors of severe recurrence of violent injury included black race (OR: 1.4 95% CI: 1.1–1.8; p = 0.018), zip code median income below national median (OR: 1.3; 95% CI 1.0–1.9; p = 0.085), and being insured by Medicaid (OR:1.5; 95% CI 1.0–2.4; p = 0.061).^39^

Other literature suggest structural risk factors such as prior incarceration lead to increased risk of injury. A study of black men who were part of a Baltimore HVIP found increased rates of hospitalization due to repeat injury in individuals with previous incarceration (OR: 8.42; CI −1,73–40.92; p <0.05) and report of using a weapon or being in a fight in the past year (OR = 2.56; CI 1.08–6.06; p <0.05).^40^ One pilot study attempted to create a clinically feasible risk index for firearm violence.^41^ The study proposed a 4-item questionnaire (SaFETy score) that evaluated: 1) serious fighting; 2) friend weapon carrying; 3) community environment; and 4) firearm threats to grade risk of future injury from firearm violence. The SaFETy score has shown potential but has not yet been externally validated or applied to individuals >24 years of age or those who do not use substances.

Finally, a recent study based on experiences from the WAP at San Francisco General Hospital proposed a clinical tool called the violent reinjury risk assessment instrument (VRRAI).^42^ The study included 11 semi-structured interviews and two focus groups with HIVP case managers and key information. The result was the development of four tiers of risk factors based on seven domains, including environment, identity, mental health, behavior, conflict, indicators of lower risk, and case management. One potential limitation is that the tool must be conducted by an individual with experiential knowledge, such as a case manager trained for the specific HVIP, rather than the emergency physicians (EP) who is most likely to determine the disposition for such patients. This requirement limits the potential for the VRRAI to be implemented widely.

The SaFETy and VRRAI are two potential clinical tools, in addition to others yet developed, that should be considered for further internal and external validation. Ultimately, feasibility and implementation studies must be considered to ensure that the risk stratification tool achieves the intended goals, including reduction of injury recidivism, associated mortality, and cost through targeted interventions.

We recognize that the ultimate outcome of such risk stratification may not prove worthwhile. Research may find that a risk stratification tool proves no more useful than clinical gestalt. Furthermore, implementation studies may find that even the lowest risk survivors of interpersonal violence still benefit from intervention. Nonetheless, we believe that in order to facilitate research that allows the growth and cost effective implementation of violence intervention programs, the development of a comprehensive risk stratification tool is a critical first step. While most EPs are exposed to penetrating trauma during their training, many are not accustomed to evaluating risk for reinjury and may benefit significantly from an evidence-based decision aid to inform their clinical decision-making. Furthermore, stratification tools and their partnership with successful HVIP may address other unmet social needs such as employment, housing, or substance use. For example, Bell et al noted that when HVIPs are associated with community partners that work to address health insurance, legal issues, and return to school, injury recidivism dropped significantly.^13^

### Resident Education

Finally, we recognize that EPs develop many of their practice patterns during residency. With this in mind, we feel it is essential that graduate medical education incorporate formalized teaching on how to consider risk factors for reinjury in clinical decision-making. The Model of the Clinical Practice of Emergency Medicine (EM model) acknowledges that residents should be able to recognize age, gender, ethnicity, barriers to communication, socioeconomic status, and other factors that affect patient management. Currently, however, there are no specific recommendations that address the role of social determinants of health in survivors of interpersonal violence.^43^ In order to cultivate future EPs who play an active role in reducing injury recidivism, we recommend that residencies: 1) educate residents on the high rates of injury recidivism and associated mortality; 2) teach residents about what risk factors, including social determinants of health and structural drivers, affect a patient’s risk of injury recidivism; 3) train residents to consider risk of injury recidivism when determining the management of a survivor of interpersonal violence; and finally 4) forge appropriate relationships across academic, non-profit, and other community stakeholders to implement strategies for violence prevention and intervention.

## LIMITATIONS

Our paper has several limitations. First, scoping reviews do not formally evaluate the quality of evidence and are thus more descriptive. We tried to reduce the bias of descriptive pitfalls by adhering closely to PRISMA-ScR standards and having several reviewers screen independently. Secondly, scoping reviews are prone to selection bias. We attempted to safeguard against selection bias by including several keywords that would capture a broad array of HVIP-related studies and adhering closely to our inclusion criteria during the review. Lastly, due to lack of formal analysis due to the heterogeneity of end points evaluated by studies collected we deemed a scoping review would be best equipped for the current landscape.

## CONCLUSION

Emergency physicians lack an evidence-based tool to help identify and manage patients at high risk for reinjury. Future research should continue to identify social and structural risk factors for injury recidivism and explore how these factors might help build a risk-stratification tool. HVIPs have shown promise in reducing physical, mental, and financial costs of reinjury however more high levels studies are needed to further understand the overall impact. Until HVIPs are more universally available, emergency physicians should be empowered through education and clinical decision aids in identifying at-risk patients who could most benefit from these services to not only reduce injury recidivism but also further explore the impact of ED and HVIP collaboration in addressing interpersonal violence.

## Figures and Tables

**Table 1:** Hospital-based violence intervention programs.

Project Name	Location	Who	Methodology	Intervention	Follow-up	Outcomes injury recidivism	Other outcomes
Borowsky et al^10^	Minneapolis-St Paul MN metropolitan area	Ages 7–15 with positive psychological screening	*Randomized control trial	Telephone based parenting education program	9 mo	Decrease in fight related injuries requiring medical care (adjusted OR 4.7; 95% CI 1.33–16.59)	Patients who received the intervention had decrease in aggressive behavior, attention problems, parent reported bullying, physical fighting, child reported victimization
Case Management - Cheng et al^22^	Baltimore, MD; Washington, DC	Ages 12–17 with peer assault injury	*Randomized control trial	Case management for 4 months	6 mo	No change (injuries requiring intervention RR 0.12 95% CI 0.01–2.42)	No significant program effect on service utilization or risk factors for injury
Caught in Crossfire Youth ALIVE 26–28	Oakland, CA	Ages 12–20 with violent injury	Retrospective case control (Becker); Retrospective cohort study (Shibru); Cost utility analysis (Chong)	Match with Crisis Intervention Specialists to provide close peer support including counseling, job placement, probation, school, housing, referrals	Up to 1 year (Becker); 18 mo (Shibru)	No change (Becker); no change (Shibru); 4% to 2.5% (Chong)	70% less likely to be arrested for any offense; 60% less likely to have any criminal involvement (Becker); cost reduction when compared to juvenile detention center costs $750,000 to $1.5 million annually (Shibru); incremental cost effectiveness for HVIP $2,941 (Chong)
Mentor Violence Intervention Prevention - Cheng et al^11^	Baltimore, MD; Washington, DC	Ages 10–15 with peer assault injury	*Randomized control trial	Youth received 6 session problem solving sessions, parents received 3 home visits	6 mo	No change (fight related injuries in last 30 days RR 0.58 95% CI 0.09–3.94)	Reduced misdemeanor activity, youth-reported aggression scores, and increasing youth self efficacy
Operation Cease Fire (now Cure Violence)^44^	New Orleans, LA	Age unknown with intentional penetrating trauma	Ecological study	Family engagement, home visits, social service needs, conflict resolution	Up 1 year	N/A	Less penetrating injuries in target zip code 20% compared to 55.6% and 93.2% in surrounding zip codes
Operation Peace Works^16^	Ventura County, CA	Age unknown gang members referred from criminal justice system	Ecological study	Mentoring, counseling, job training, education/employment	Up to 3 year	N/A	Decrease in gang assaults (−16% P<0.001); assaults with firearms (−32% p<0.001); and homicides (−47% p=0.05)
Project Prescription Hope^12^,^13^	Indianapolis, IN	Ages >18 with interpersonal violent injury	*Prospective cohort trial (Gomez); retrospective chart review (Bell)	Tailored service plan and referred community services. Goals include 1) health insurance; 2) PCP; 3) full time employment or return to school; 4) resolve legal issues	Variable	Violent injury recidivism rate 8.7% to 2.9% (Gomez); 4.4% recidivism rate from 2009–2016 among individuals part of program (Bell)	>half of new violence related injuries outside of HVIP affiliated trauma center (Bell)
Project UJIMA^45^	Milwaukee, WI	Ages 10–18 with interpersonal violent injury	Retrospective cohort	Home visits, mental health services, youth activities	Up to 1 year	1% injury recidivism (no comparison)	N/A
SaferFlint Teens^19^–^21^	Flint, MI	Ages 14–18 that report alcohol and violence in past year	*Randomized control trial	35-minute BI delivered by computer or therapist	3 mo, 6 mo, 12 mo	N/A	Significant reductions in positive attitudes for alcohol use and violence and increase in self efficacy related to violence - at 0 months and 3 months (Cunningham 2009). Significant reduction in peer aggression in therapist group only at 12 months (Cunningham 2012)
Turning Point^14^	Philadelphia, PA	Age >18 with GSW or stab wound and admitted to hospital	Prospective randomized trial	Social work; outpatient case manager, psychiatric assessment, watch trauma bay video, meet GSW survivor	Up to 2 year	N/A	50% reduction in aggressive response to shame, 29% reduction in comfort with aggression, 19% reduction in overall proclivity toward violence
VCU Bridging the Gap^29^	Richmond, VA	Ages 10–24 with intentional injury	Randomized control trial	Brief hospital based intervention + intensive community-based case management services	6 mo	No change	Better hospital service utilization, CMS, and risk factor reduction with additional case management services
Violence Intervention Project^15^	Baltimore, MD	Ages >18 with prior violent injury and involvement in criminal justice system	Randomized control trial	Culturally sensitive violence intervention program including case/social worker and parole officer	Up to 2 year	35% (control) vs 5% (intervention)	Control group 3x more likely arrested for violent crime, 2x more likely convicted of any crime, 4x more likely convicted of violent crime with potential cost savings $1.5 million
Within Our Reach^23^–^25^	Chicago, IL	Ages 10–24 with interpersonal violent injury	Randomized control trial	Case management for 6 months	6 mo, 12 mo	Repeat victim of violence 20.3% (control) vs 8.1% (intervention)	Return ED visit control 7.4% vs 6.5% intervention. No change in self reported arrests, state reported reinjuries via trauma register, or state reported incarcerations
Wraparound Project^17^,^18^,^30^,^46^	San Francisco, CA	Ages 10–30 with intentional injury and determined to be at high risk for reinjury based on structured screening process	Longitudinal observational study (Julliard 2016); Cost utility analysis (Julliard 2015); Retrospective cohort study (Smith 2013)	Intensive culturally competent case management	6–12 mo	8% (historical) to 4% (Julliard 2016); 16% (historical) vs 4% (Smith 2013)	VIP costs less than having no VIP; VIP yields health benefits (24 QALYs) and savings ($4100) if implemented for 100 individuals OR $6000 saved per patient over 5 years (Julliard 2015); most successful when meeting needs with mental health and employment (Smith 2013)
Zatzick et al^47^	Seattle, WA	Ages 12–18 that survived intentional and unintentional injuries	Randomized control trial	Collaborate care intervention with motivational interviewing, medication and cognitive behavioral therapy targeting PTSD and depressive symptoms	2 mo, 5 mo, 12 mo	N/A	Decrease in carrying weapon 7.3% intervention vs 21.3% control patients 12 months after injury
James et al^48^	Boston, MA	ED patients >18yr enrolled in Violence intervention advocacy program (VIAP)	Exploratory Qualitative study	Structural interviews that underwent content analysis with grounded theory for identified themes of VIAP effectiveness	Active enrollment in VIAP	Participants described positive, life-changing behaviors on their journey to healing through connections to caring, supportive adults. Information gained	N/A

*RR*, relative risk; *OR*, odds ratio; *CI*, confidence interval; *mo*, month; *PCP*, primary care provider; *HVIP*, hospital-based violence intervention program; *GSW*, gunshot wound; *CMS*, Centers for Medicare & Medicaid; *QUALYS*, quality adjusted life years.
